# Factors associated with suicidal ideation among medical residents in Tehran during the COVID-19 pandemic: A multicentric cross-sectional survey

**DOI:** 10.1371/journal.pone.0300394

**Published:** 2024-03-15

**Authors:** Fahimeh Saeed, Elaheh Ghalehnovi, Mahdieh Saeidi, Neda Ali beigi, Mohsen Vahedi, Mohammadreza Shalbafan, Leila Kamalzadeh, Ali Nazeri Astaneh, Amir Hossein Jalali Nadoushan, Sheikh Shoib

**Affiliations:** 1 Psychosis Research Center, University of Social Welfare and Rehabilitation Sciences, Tehran, Iran; 2 Razi Vaccine and Serum research Institute, Karaj, Iran; 3 Research Center for Addiction and Risky Behaviors, Iran University of Medical Sciences, Tehran, Iran; 4 Department of Biostatistics and Epidemiology, Pediatric Neurorehabilitation Research Center, University of Social Welfare and Rehabilitation Sciences, Tehran, Iran; 5 Department of Psychiatry, Mental Health Research Center, Psychosocial Health Research Institute (PHRI), School of Medicine, Iran University of Medical Sciences, Tehran, Iran; 6 Department of Psychiatry, Geriatric mental health research center, School of Medicine, Iran University of Medical Sciences, Tehran, Iran; 7 Department of Health Services, Srinagar, Kashmir, India; University of Huelva: Universidad de Huelva, SPAIN

## Abstract

**Background:**

The mental health of medical residents, challenged by their intensive training, is of utmost concern. In light of reported suicides among Iranian medical residents in 2021, this study investigates the factors behind suicidal ideation among medical residents during the COVID-19 pandemic in Tehran.

**Methods:**

This study conducted a cross-sectional online survey among medical residents in various specialties in Tehran, Iran, amidst the COVID-19 pandemic. Suicidal ideation was assessed using the Beck Scale for Suicidal Ideation (BSSI), while depression, anxiety, and stress were measured using the DASS-21. It also collected demographic and clinical data from the participants. The data were analyzed using descriptive statistics, the Chi-square test, and multiple linear regression to examine the prevalence and determinants of suicidal ideation among medical residents.

**Results:**

The study enrolled 353 medical residents and found that 34.3% of them had suicidal ideation, with 10.2% indicating a high risk. The study also found high levels of depression, anxiety, and stress among the participants. The variables that significantly predicted suicidal ideation were depression, history of alcohol/substance use, personal history of suicide attempts, history of self-mutilation, family history of suicide attempts, number of shifts in a month, death of close persons because of COVID-19, and income. Depression was the strongest predictor of suicidal ideation.

**Conclusion:**

These findings underscore the urgent need for effective interventions and support systems to address the mental health needs of medical residents in Iran. The strategies should prioritize destigmatizing mental health, promoting access to mental health services, fostering a supportive training environment, and enhancing income opportunities.

## Introduction

The mental well-being of medical residents is a critical concern due to the demanding and challenging nature of their training, which can have profound implications for patient care [1]. Medical residency represents a pivotal phase in physicians’ careers, characterized by long working hours, sleep deprivation, heightened stress levels, social deprivation, and exposure to emotionally taxing circumstances. These factors can cause considerable psychological distress and may increase the risk of developing psychiatric disorders [2].

Research conducted in low- and middle-income countries has shown a notable prevalence of depression and anxiety among residents in various specialized fields, ranging from 11% to 65% [3–7]. While residents in these countries face additional challenges, such as limited healthcare resources, low income, and disparities in healthcare distribution [4], it is crucial to recognize that high rates of mental health problems in medical residents are not exclusive to developing countries. Studies conducted in high-income countries have also reported similarly elevated rates of depressive and anxiety disorders among medical residents, ranging from 7% to 43% [8–11]. A comprehensive meta-analysis incorporating data from 54 studies worldwide revealed an overall prevalence of depressive symptoms of 28.8% among 17,560 resident physicians (95% CI, 25.3%-32.5%) [12]. These rates significantly exceed the lifetime prevalence of depression in the general adult population worldwide, which stands at 5%, as verified by the World Health Organization (WHO) [13].

Depression, particularly when comorbid with anxiety disorders, can lead to severe outcomes such as suicidal thoughts and behaviors [14]. Several studies have documented the occurrence of suicidal ideation among medical residents, with prevalence rates ranging from 4 to 35 percent [15–18]. According to the Accreditation Council for Graduate Medical Education (ACGME), suicide is the second leading cause of resident deaths in the USA, with the majority of suicides occurring during the first two years of training [19]. Disturbingly, reports from India indicate 105 suicide-related deaths among residents between 2010 and 2019, with academic stress and harassment identified as significant risk factors [20,21]. Various factors have been proposed to explain the high suicide rate among residents, including long and unpredictable work shifts, sleep deprivation, burnout and impaired cognitive performance, high-stress situations (e.g., life and death emergencies), and easy access to means of self-harm [15,17,22].

The predictors of suicidal thoughts and behaviors among medical residents may vary across different regions, cultures, and contexts. Prior to the COVID-19 pandemic, specific data pertaining to suicide rates among Iranian medical residents was scarce. A comprehensive review and meta-analysis conducted in 2016 indicated a prevalence of 11.1% (95% CI, 9.0% to 13.7%) in suicidal ideation among medical students [23]. Recent years, however, have witnessed a concerning escalation in suicide cases among medical residents. Reports from various Iranian news outlets have highlighted that between 2019 and June 2021, there were 15 documented suicide cases involving physicians and medical residents [24]. This trend is particularly alarming, with a significant number of these cases occurring in a relatively short timeframe, indicating an increase in such incidents compared to historical data. Contrary to the anticipated decrease in suicide rates among medical professionals following the subsidence of the COVID-19 pandemic, the reality has been a continued prevalence of these tragic events [25]. Contributing factors to this persistent issue include heightened workloads, substantial patient care duties, financial pressures, societal stigma surrounding mental health issues, and inadequate support structures within the healthcare system [24,26,27]. However, a comprehensive assessment of the dimensions of this issue is yet to be conducted. Hence, this study aimed to shed light on factors associated with suicidal ideation among medical residents in Iran. Understanding the unique challenges medical residents face in Iran is crucial for developing effective interventions and support systems to address their mental health needs.

## Methods

### Study design

This cross-sectional survey aimed to explore the factors associated with suicidal ideation among medical residents from different specialties in Tehran, the capital of Iran, during the COVID-19 pandemic. The survey used a combination of standardized scales and demographic questions to measure the variables of interest.

### Setting

The study was conducted online due to COVID-19 related social distancing measures, ensuring easy access for the majority of medical residents. The online questionnaire was distributed through WhatsApp groups to medical university residents in Tehran. It was available 24/7 from Jan. 1, 2022, to Feb. 28, 2022, and concluded upon reaching the desired sample size.

### Participants

The study included medical residents from various specialized fields across Tehran Medical Universities, namely Tehran University of Medical Sciences (TUMS), Shahid Beheshti University of Medical Sciences (SBUMS), Iran University of Medical Sciences (IUMS), AJA University of Medical Sciences and University of Social Welfare and Rehabilitation Sciences (USWR), representing different stages of residency training. According to recent information from the Iranian Ministry of Health, and Medical Education, these universities together accommodate around 4,000 medical residents, which accounts for about one-third of the nation’s total number of medical residents [28]. Based on their field of practice, they were categorized into three groups: procedural specialties (including general surgery, neurosurgery, gynecology, emergency medicine, otolaryngology, urology, Orthopedics, and ophthalmology), clinical specialties (compromising internal medicine, psychiatry, neurology, pediatrics, radio-oncology, sports medicine, social medicine, and cardiology), and diagnostic specialties (encompassing radiology, pathology, and nuclear medicine).

The introductory page of the questionnaire explicitly outlined the study’s purpose. The inclusion criteria were detailed on the second page, which required respondents to confirm their status as residents currently enrolled at the specified universities in Tehran and their consent to participate in the study. Only those who answered affirmatively to these initial queries were granted access to the full questionnaire, ensuring that the study focused on a specific and relevant participant group. The exclusion criteria for this study comprised individuals who did not provide informed consent to participate in the research and those who were absent from the teaching hospital for three months preceding the study. In the absence of existing research, we used a conservative 50% prevalence rate for our sample size calculation, specifically referring to suicidal ideation among medical residents to encompass both low and high suicide risk levels. Employing a statistical power analysis with a 5% margin of error and a 95% confidence level, we determined that a sample size of 351 participants was optimal. A convenience sampling method was utilized in this study.

### Measures

The online questionnaire comprised a total of 64 items encompassing various domains, including demographic data, specialty, year of residency, suicidal ideation, having children, living arrangements, accommodation, history of psychiatric disorders, alcohol/substance use, family history of suicide attempts, personal history of suicide attempts, history of self-mutilation, number of shifts in a month, COVID-19 work exposure, death of close persons because of COVID-19, use of mental health services during COVID-19 pandemic, income, and components of the Beck Scale for Suicidal Ideation (BSSI), as well as items from the Depression, Anxiety, and Stress Scale-21 (DASS-21).

#### Beck Scale for Suicidal Ideation (BSSI)

The Beck Scale for Suicidal Ideation (BSSI) is a widely recognized and valuable tool for assessing suicidal ideation and behavior. It was developed in 1979 by Aaron T. Beck and consists of a 19-item self-report questionnaire that measures three dimensions of suicidal ideation: severity, frequency, and intent. Each item is rated on a scale of 0 to 2, indicating the absence, mild presence, or strong presence of specific suicidal thoughts. The total BSSI score ranges from 0 to 38 [29].

The first five items of the BSSI can be used to identify individuals with suicidal thoughts. If patients score zero on the Active Suicidal Desire item (#4) or the Passive Suicidal Desire item (#5), indicating no active or passive intention to die, they skip the remaining 14 items. Otherwise, the remaining 14 items are rated. Patients with active or passive ideation about killing themselves are considered suicide ideators [30].

There is no universally defined cut-off score for BSSI to determine suicide risk levels; however, studies have proposed various criteria based on empirical evidence and clinical judgment. In this regard, scores of 0 on all 19 BSSI items indicate no risk of suicide. Scores ranging from 0 to 5 indicate low risk, while scores of 6 and above indicate high risk [31].

Extensive research has substantiated the robust psychometric properties of the BSSI, demonstrating high internal consistency (Cronbach’s alpha coefficients between 0.89 and 0.96) and satisfactory test-retest reliability (correlation coefficients above 0.80) [32]. In the study conducted by Esfahani et al., the Persian version of the Beck Scale for Suicidal Ideation (BSSI) utilized in this research exhibited substantial internal consistency, as indicated by Cronbach’s alpha values ranging from 0.829 to 0.837. Furthermore, this version of the BSSI demonstrated significant correlations with depressive symptoms and revealed a unidimensional factor structure [33].

#### Depression, Anxiety, and Stress Scale-21 (DASS-21)

The DASS-21 is a screening instrument designed to assess symptoms of depression, anxiety, and stress experienced in the week leading up to the evaluation. It consists of three subscales (depression, anxiety, and stress), each comprising seven items. The final score for each subscale is calculated by summing the scores of the corresponding items, which are rated on a scale of 0 (does not apply to me at all) to 3 (applies to me very much or most of the time) [34]. The DASS-21 has demonstrated good internal consistency and reliability, as validated in various languages and populations [35]. The Persian DASS-21 study demonstrated high internal consistency (Cronbach’s alpha: anxiety 0.79, stress 0.91, depression 0.93), robust test-retest reliability (0.740–0.881), and satisfactory model fit in confirmatory factor analysis (RMSEA 0.078, CFI 0.917), with ICCs from 0.75 to 0.86 confirming reliability in a nurse sample [36].

### Statistical analysis

The data were analyzed using SPSS software version 25. Descriptive statistics such as frequency and percentage were used to summarize the characteristics of the participants and their scores on the BSSI and the DASS-21. A Chi-square test was used to examine the association between suicidal ideation and other clinical and demographic variables. Multiple linear regression analysis was employed to determine the predictors of suicidal ideation, based on variables significantly correlated in the Chi-square test, and to adjust for potential confounders in our examination of suicidal ideation among medical residents. The level of significance was set at 0.05 for all tests. Cases with missing data were entirely excluded from the analysis.

### Ethical considerations

The research protocol received approval from the Ethics Committee of the University of Social Welfare and Rehabilitation Sciences (Code: IR.USWR.REC.1400.226) and was conducted in adherence to the principles outlined in the Declaration of Helsinki. The questionnaire clearly explained the study’s objectives, and participants were required to provide online written informed consent. No personally identifiable information was collected, and authors did not have access to information that could identify individual participants during or after data collection. However, participants could provide their email addresses if they were interested in receiving study results. For individuals identified as being at moderate to high risk of suicide, we sent proactive messages to their respective email addresses, urging them to seek counseling with a volunteer psychiatrist coauthor who was available to assist.

## Results

Out of the total of 359 residents who responded to the survey, six residents did not complete it. As a result, the final number of participants included in the study was 353. The analysis included data from all these participants. The results show that most of the participants were female, aged 30–34 years, married, clinical specialists, in their first year of residency, without children, living with relatives, renting, or owning a home, without a history of psychiatric disorders, alcohol/substance use, family history of suicide attempts, personal history of suicide attempts or self-mutilation. The average number of shifts in a month was 0–7, and the level of COVID-19 work exposure was moderate. Most of the participants had a history of COVID-19 infection but were not admitted due to COVID-19 infection or the death of close persons because of COVID-19. About one-third of the participants used mental health services during the COVID-19 pandemic and had an income of less than 6 million Tomans per month (≈200 €).

The BSSI scores from our study revealed that the majority of participants did not show suicidal ideation, while a notable portion exhibited low and high-risk suicidal thoughts. Additionally, the prevalence of depression, anxiety, and stress was significant among participants, with a substantial number experiencing moderate to very severe levels of these conditions. Further demographic and clinical characteristics can be found in Table 1.

**Table 1:** Demographic and clinical characteristics of participants.

Characteristic		Frequency	Percent
**Gender**	Female	268	75.9
	Male	85	24.1
**Age**	25–29	125	35.4
	30–34	177	50.1
	35–39	33	9.3
	40 years and above	18	5.1
**Marital status**	Married	205	58.1
	Single	132	37.4
	Divorced	16	4.5
**Specialty**	Procedural	84	23.8
	Clinical	239	67.7
	Diagnostic	30	8.4
**Year of residency**	1	111	31.4
	2	83	23.5
	3	84	23.8
	4 and above	75	21.2
**Having children**	No	298	84.4
	Yes	55	15.6
**Living arrangement**	Spouse	71	20.1
	Relatives	247	70.0
	Peers	35	9.9
**Accommodation**	Dormitory	32	9.1
	Rental home	157	44.5
	Personal home	164	46.5
**History of psychiatric disorders**	No	224	63.5
	Yes	129	36.5
**History of alcohol/substance use**	No	273	77.3
	Yes	80	22.7
**Family history of suicide attempts**	No	302	85.6
	Yes	51	14.4
**Personal history of suicide attempts**	No	334	94.6
	Yes	19	5.4
**History of self- mutilation**	No	235	94.9
	Yes	18	5.1
**Number of shifts in a month**	0–7	175	49.6
	8–14	161	45.6
	15 and above	17	4.8
**COVID-19 Work Exposure**	High exposure *(Working in COVID-19 wards or respiratory emergency)*	89	25.2
	Moderate exposure *(Occasional visits or consultations in the COVID-19 ward)*	152	43.1
	No exposure	112	31.7
**History of COVID-19 infection**	No	92	26
	Yes	261	74
**History of admission due to COVID-19 infection**	No	336	95.2
	Yes	17	4.8
**Death of Close Persons because of COVID-19**	No	201	56.9
	Yes	152	43.1
**Use of Mental Health Services during COVID-19 Pandemic**	No	246	69.7
	Yes	107	33
**Income per month**	Less than 6 million Tomans (≈200 €)	226	64
	6–9 million Tomans (≈ 200–300 €)	101	28.6
	More than 9 million Tomans (≈300 €)	26	7.4
**BSSI**	No suicidal ideation	232	65.7
	Low risk suicidal ideation	85	24.1
	High risk suicidal ideation	36	10.2
	Overall prevalence	121	34.3
**Depression**	No depression	108	30.6
	Mild	45	12.7
	Moderate	110	31.2
	Severe	33	9.3
	Very severe	57	16.1
**Anxiety**	No anxiety	130	36.8
	Mild	42	11.9
	Moderate	112	31.7
	Severe	31	8.8
	Very severe	38	10.8
**Stress**	No stress	125	35.4
	Mild	68	19.3
	Moderate	77	21.8
	Severe	65	18.4
	Very severe	18	5.1

Note: Percentages are based on the total number of participants (N = 353). BSSI: Beck Scale for Suicide Ideation.

The association between suicidal ideation and other clinical and demographic variables was examined using Pearson Chi-Square test. Table 2 presents the results of the analysis.

**Table 2:** Association between suicidal ideation and other clinical and demographic variables.

Variables		No suicidal ideation	Low risk suicidal ideation	High risk suicidal ideation	Pearson Chi-Square Value	df	*P* value
**Gender**	Female	173(64.6%)	69(25.7%)	26(9.7%)	1.786	2	.410
	male	59(64.1%)	16(18.8%)	10(11.8%)			
**Age**	25–29	86(68.8%)	26(20.8%)	13(10.4%)	7.321	4	.560
	30–34	115(65%)	46(26%)	16(9%)			
	35–39	19(57.6%)	9(27.3%)	5(15.1%)			
	40 years and above	12(66.7%)	4((22.2%)	2((11.1%)			
**Marital status**	Married	142(69.3%)	45(22%)	18(8.8%)	7.149	4	.128
	Single	84(63.7%)	33(25%)	15(11.4%)			
	Divorced	6(37.5%)	7(43.8%)	3(18.7%)			
**Specialty**	Procedural	46(54.8%)	28(33.3%)	10(11.9%)	6.658	4	.155
	Clinical	164(68.7%)	51(21.3%)	24(10%)			
	Diagnostic	22(73.3%)	6(20%)	2(6.7%)			
**Year of residency**	1	77(69.4%)	25(22.5%)	9(8.1%)	5.081	6	.533
	2	50(60.2%)	24(29%)	9(10.8%)			
	3	51(60.7%)	21(25%)	12(14.3%)			
	4 and above	54(72%)	15(20%)	6(8%)			
**Having children**	No	196(65.8%)	71(23.8%)	31(10.4%)	.130	2	.937
	Yes	36(65.4%)	14(25.5%)	5(9.1%)			
**Living arrangement**	Spouse	44(62%)	17(24%)	10(14%)	2.298	4	.681
	Relatives	167(67.6%)	58(23.5%)	22(8.9%)			
	Peers	21(60%)	10(28.6%)	4(11.4%)			
**Accommodation**	Dormitory	21(65.6%)	7(21.9%)	4(12.5%)	3.116	4	.539
	Rental home	98(62.5%)	39(24.8%)	20(12.7%)			
	Personal home	113(68.9%)	39(23.8%)	12(7.31%)			
**History of psychiatric disorders**	No	161(45%)	48(15%)	15(4%)	12.690	2	**.002**
	Yes	71(20%)	37(10%)	21(6%)			
**History of Alcohol/substance use**	No	191(54%)	55(16%)	27(8%)	11.146	2	**.004**
	Yes	41(12%)	30(8%)	9(2%)			
**Family history of suicide attempts**	No	209(59%)	65(19%)	28(8%)	11.291	2	**.004**
**Personal history of suicide attempts**	Yes	23(6%)	20(5%)	8(3%)			
	No	226(64%)	81(22%)	27(8%)	30.843	2	**.001**
	Yes	6(2%)	4(1%)	9(3%)			
**History of self- mutilation**	No	228(64%)	77(22%)	30(9%)	18.682	2	**.001**
	Yes	4(1%)	8(3%)	6(2%)			
**Number of shifts in a month**	0–7	126(72%)	35(20%)	14(8%)	8.941	4	**.049**
	8–14	99(61.5%)	43(26.7%)	19(11.8%)			
	15 and above	7(41.2%)	7(41.2%)	3(17.6%)			
**COVID-19 Work Exposure**	Moderate exposure (Occasional visits or consultations in the COVID-19 ward)	109(31%)	29(8%)	14(4%)	13.085	4	**.011**
	High exposure (Working in COVID-19 wards or respiratory emergency)	45(13%)	30(8%)	14(4%)			
	No exposure	78(22%)	26(7%)	8(3%)			
**History of COVID-19 infection**	No	61(17%)	23(6%)	8(3%)	.326	2	.850
	Yes	171(48%)	62(18%)	28(8%)			
**History of admission due to COVID-19 infection**	No	223(63%)	80(22%)	33(9%)	1.627	2	.443
	Yes	9(3%)	5(2%)	3(1%)			
**Death of Close Persons because of COVID-19**	No	153(43%)	37(10%)	11(3%)	24.13	2	**.001**
	Yes	79(23%)	48(14%)	25(7%)			
**Use of Mental Health Services during COVID-19 Pandemic**	No	166(47%)	63(18%)	17(5%)	9.77	2	**.008**
	Yes	66(19%)	22(6%)	19(5%)			
**Income per month**	Less than 6 million Tomans (≈200 €)	141(40%)	57(16%)	28(8%)	9.200	4	**.049**
	6–9 million Tomans (≈200–300 €)	74(22%)	19(5%)	4(1%)			
	More than 9 million Tomans(≈300 €)	19(5%)	7(2%)	2(1%)			
**Depression**	No depression	102(29%)	6(2%)	0	146.128	8	**.001**
	Mild	37(10%)	7(2%)	1(1%)			
	Moderate	63(18%)	43(13%)	4(1%)			
	Severe	14(4%)	13(4%)	6(2%)			
	Very severe	16(4%)	16(4%)	25(6%)			
**Anxiety**	No anxiety	107(30%)	21(6%)	2(0.5%)	81.342	8	**.001**
	Mild	29(8%)	11(3%)	2(0.5%)			
	Moderate	61(17%)	37(10%)	14(4%)			
	Severe	22(6%)	4(1%)	5(2%)			
	Very severe	13(4%)	12(4%)	13(4%)			
**Stress**	No stress	105(30%)	18(5%)	2(0.5%)	81.342	8	**.001**
	Mild	46(14%)	20(6%)	2(0.5%)			
	Moderate	49(14%)	21(6%)	7(2%)			
	Severe	25(7%)	24(7%)	16(4%)			
	Very severe	7(2%)	2(0.5%)	9(3%)			

Significant values are in bold (P<0.05).

The analysis showed that factors such as gender, marital status, type of specialty, residency year, having children, living arrangements, type of accommodation, history of COVID-19 infection, and history of hospitalization due to COVID-19 were not statistically significant in their association with suicidal ideation.

History of psychiatric disorders, alcohol/substance use, family history of suicide attempts, personal history of suicide attempts, history of self-mutilation, and number of shifts in a month were all significantly associated with suicidal thoughts. COVID-19 work exposure was also significantly associated with suicidal ideation, with high exposure being more prevalent among those with high-risk suicidal ideation than those with no or low risk suicidal ideation.

Death of close persons because of COVID-19 and use of mental health services during the COVID-19 pandemic were significantly associated with suicidal ideation, with higher use among those with high risk than those with no or low risk suicidal ideation.

Income was marginally significantly associated with suicidal ideation, with lower income being more prevalent among those with high risk than those with no or low risk suicidal ideation.

Depression, anxiety, and stress were all significantly associated with suicidal ideation, with higher levels of depression, anxiety, and stress being more prevalent among those with high risk than those with no or low risk suicidal ideation.

A linear regression analysis was conducted to identify the predictors of suicide among the variables studied. Based on the Chi-square test results, only the statistically significant variables, and the continues score of suicide ideation were included in the regression model to examine the amount of variance explained (Table 3).

**Table 3:** Regression coefficients of suicide ideation* and dependent variables.

Model		Unstandardized Coefficients		Standardized Coefficients	t	*p* value
		B	Std. Error	Beta		
	**(Constant)**	-.246	.482		-.510	.611
	**History of psychiatric disorders**	-1.007	2.462	.031	.409	.683
	**History of** **Alcohol/substance use**	.197	.094	.177	2.098	**.038**
	**Family history of suicide attempts**	3.149	1.187	.136	2.654	**.008**
	**Personal history of suicide attempts**	4.161	2.073	.147	2.007	**.047**
	**History of self- mutilation**	7.097	1.926	.191	3.685	**.001**
	**Number of shifts in a month**	.230	.99	.127	2.325	**.021**
	**COVID-19 Work Exposure**	1.930	1.081	0.203	1.176	0.75
	**Death of Close Persons because of COVID-19**	1.916	.835	.117	2.295	**0.02**
	**Use of Mental Health Services during COVID-19 Pandemic**	1.360	.928	0.75	1.466	0.144
	**Income**	-.155	.071	-.175	-2.185	**.031**
	**Depression**	.561	.113	.604	4.964	**.001**
	**Anxiety**	.144	.126	.120	1.150	.253
	**Stress**	. 062	.153	.053	-.402	.688

Significant values are in bold (P<0.05)

*The continuous score of suicide ideation was used in analyses.

The analysis revealed that suicide ideation was significantly predicted by a history of alcohol/substance use, family history of suicide attempts, personal history of suicide attempts, history of self-mutilation, number of shifts in a month, death of close persons because of COVID-19, income, and depression (all p-values < 0.05). All these variables, except for income, had positive associations with suicide ideation, indicating that higher levels or categories of these variables corresponded to higher levels of suicide ideation. The most influential predictor was depression (Beta = 0.604), followed by COVID-19 work exposure (Beta = 0.203) and a history of self-mutilation (Beta = 0.191).

## Discussion

This study examined the factors associated with suicidal ideation among medical residents in Iran during the third and fourth waves of the COVID-19 pandemic. The findings revealed that a significant proportion of participants (34.3%) reported having suicidal ideas, with 10.2% indicating high-risk suicidal thoughts. These rates were higher than those reported in the general population of Iran (12.7–14%) and in medical students (7–26%) in previous studies [37–40]. The prevalence of suicidal thoughts among medical residents in this study also surpassed the rates reported in recent studies conducted in other countries [10,41,42].

The study further explored the levels of depression, anxiety, and stress experienced by the participants. The findings revealed that more than 50% of the medical residents reported moderate to severe symptoms. This aligns with the results of a recent study conducted on 150 medical residents from Tehran University of Medical Sciences in Iran, where 23% of residents reported severe to highly severe depression, 24.9% reported severe to highly severe anxiety, and 33.8% reported severe to highly severe stress [6]. Notably, these percentages significantly surpass those reported in pre-pandemic studies. For instance, a study conducted in 2007 reported depression in only 15.6% of medical residents in Tehran [43], while another study in 2010 found that 92% of Iranian medical residents did not experience anxiety symptoms [44]. Consequently, the high prevalence of depression and anxiety observed in our study raises concerns. Several factors contribute to this phenomenon. The COVID-19 pandemic is a significant factor, as it has posed unprecedented challenges and stressors for healthcare workers, especially those in training [45]. The socio-economic challenges faced by Iran in recent years may have also impacted the well-being of healthcare workers [46]. Moreover, mental health stigma in the medical community discourages residents from seeking help and discussing their struggles openly. The fear of being perceived as weak hinders them from seeking professional support [47]. Additionally, the demanding nature of their training and long working hours limit their ability to prioritize mental well-being or access mental health services [8].

Among the demographic and personal factors assessed, a history of psychiatric disorders, alcohol/substance use, family history of suicide attempts, personal history of suicide attempts, and history of self-mutilation were positively related to suicidal ideation. These factors indicate higher vulnerability and lower resilience in coping with stress and have been previously identified as risk factors for suicidal behavior across various populations [7,8,10,15]. Medical residents with such characteristics require enhanced attention and support from mental health professionals and institutions [20,48]. Contrary to previous studies conducted in the general population of Iran [37,38,49], demographic factors such as gender, marital status, number of children, living arrangement, and accommodation did not show a significant relationship with suicidal ideation in our study. This suggests potential differences in the risk factors for suicidal ideation between the general population and medical residents in Iran. Our study did not identify a significant variation in the frequency of suicidal thoughts among different age groups of the participants. However, it is essential to acknowledge that the age range of the participating residents was relatively narrow and that this age range has been consistently identified as having the highest susceptibility to suicide in prior studies conducted among the general population [38,49,50].

Regarding work-related factors, our study found a positive correlation between the number of shifts per month, COVID-19 work exposure, and suicidal ideation among medical residents. This observation necessitates a comparison of working hours for medical residents in Iran with global standards. Notably, Iranian medical residents often have longer working hours compared to their counterparts in countries like the US, Canada, and Turkey [51]. The ACGME, for instance, limits resident work hours to 80 per week, restricts continuous work to 24 hours, and mandates significant rest periods between shifts and days off. In contrast, Iranian residents frequently work beyond these limits, reflecting the intense workload and stress they face, which is further exacerbated during pandemic conditions [51,52]. This situation, coupled with high responsibility and exposure to traumatic events, has been linked to increased mental health problems and suicidal ideation in healthcare workers [45,53]. Our study’s lack of significant variation in suicidal ideation across residency years contrasts with other research showing notable differences. For instance, a study on ACGME-Accredited Programs’ residents found higher suicide rates early in residency, especially during specific academic months [19]. This parallels a study of 740 interns, where a 370% increase in suicidal ideation was noted in the initial three months of internship, highlighting the stress associated with transitions in medical training [54]. Several factors specific to our study context and methodology could explain this discrepancy. Firstly, the uniformly high stress levels across all residency years in our study, possibly exacerbated by the COVID-19 pandemic, may have obscured any year-specific variations. Additionally, cultural factors in Iran, such as potential underreporting due to mental health stigma, could have influenced residents’ reporting of mental health issues, masking any differences in suicidal ideation [55]. Lastly, the composition and size of our sample, particularly the subgroups for each residency year, might not have been sufficient to detect significant differences. Although our study did not find a significant difference in suicide risk across various medical specialties, earlier research has indicated a heightened risk in specific fields such as anesthesiology, general surgery, and internal medicine [53]. This lack of disparity in our findings could potentially be related to the size and specialty distribution of our sample. The representation of different specialties in our study might not have been sufficiently comprehensive to detect nuanced variations in suicide risk.

The findings of this study also indicated a positive association between suicidal thoughts and the loss of close persons because of COVID-19. This factor indicates the personal loss and grief some medical residents may experience during the pandemic, affecting their mental health and well-being. Bereavement resulting from COVID-19 has been associated with a heightened risk of depression, anxiety, post-traumatic stress disorder, and complicated grief [56].

Among the economic factors, income was negatively related to suicidal ideation. This factor reflects the financial difficulties that some medical residents may face, which may add to their stress [52].

Depression emerged as the primary predictor of suicidal ideation among medical residents, highlighting the profound emotional distress that some individuals in this group may endure, leading to impaired functioning and diminished quality of life. Extensive evidence has consistently shown that psychiatric disorders, particularly depression, substantially elevate the risk of suicide across diverse populations, including healthcare workers [37,42,57]. However, many training programs have faced challenges in identifying and offering treatment to medical students and residents at risk [58]. These findings underscore the significance of screening for depression and delivering effective treatment to medical residents experiencing depression.

The study has several limitations that should be acknowledged. First, the study used a cross-sectional design, which limits the causal inference between the variables. Longitudinal studies are needed to examine the temporal relationship between suicidal thoughts and other factors among medical residents. Second, the study relied on self-report measures, which may be subject to recall and social desirability biases. Objective measures such as clinical interviews are needed to validate the self-report measures. Third, the use of convenience sampling in our study may not have adequately represented all medical specialties, limiting comparative analysis. Furthermore, the focus on medical residents from a single city narrows the generalizability of our results. Representative samples from various regions or cities are necessary to understand cross-cultural variations in suicidal thoughts among medical residents. The fourth limitation of our study is the omission of additional risk factors that could potentially influence the outcomes as confounding factors. For instance, we did not assess the impact of personality traits, marital discord, conflicts with family or colleagues, sleep quality, chronic medical conditions, and the potential protective role of social support and religious beliefs. These factors have been identified in previous research as potential contributors to mental health outcomes [59–61] and could have provided a more comprehensive understanding of the associations observed in our study. Additionally, the study did not verify respondent authenticity, introducing potential uncertainty in the findings and emphasizing the need for future research to confirm the reliability of data. In the realm of future research, the inclusion of institutional reviews conducted in collaboration with medical students’ union councils and medical specialty residents’ associations within individual universities holds great promise. These reviews can serve as invaluable tools for gaining a comprehensive understanding of the multifaceted landscape encompassing the needs, challenges, and overall satisfaction levels of medical residents.

## Conclusions

This study casts vital light on the urgent mental health issues among Iranian medical residents during the COVID-19 pandemic, providing new insights into the prevalence and drivers of suicidal ideation in a previously underexplored context. The high rates of suicidal thoughts found in this study highlight the urgent need for targeted mental health interventions, particularly focusing on depression as a key predictor. Addressing these challenges through effective depression screening and treatment is crucial for both improving the mental health and sustaining the professional effectiveness of these vital healthcare workers.

## Supporting information

S1 Checklist STROBE statement—Checklist of items that should be included in reports of observational studies. (DOCX)

## References

[1] Pereira-Lima K, Mata DA, Loureiro SR, Crippa JA, Bolsoni LM, Sen S. Association between Physician Depressive Symptoms and Medical Errors: A Systematic Review and Meta-analysis. JAMA Network Open. 2019. doi: 10.1001/jamanetworkopen.2019.16097 31774520 PMC6902829

[2] Baker K, Sen S. Healing medicine’s future: Prioritizing physician trainee mental health. AMA J Ethics. 2016;18. doi: 10.1001/journalofethics.2016.18.6.medu1-1606 27322994 PMC5503146

[3] Demir F, Ay P, Erbaş M, Özdil M, Yaşar E. The prevalence of depression and its associated factors among resident doctors working in a training hospital in Istanbul. Turk Psikiyatri Dergisi. 2007;18. 17364266

[4] Yousuf A, Ishaque S, Qidwai W. Depression and its associated risk factors in medical and surgical post graduate trainees at a teaching hospital: A cross sectional survey from a developing country. J Pak Med Assoc. 2011;61. 22356028

[5] Kwok C. Depression, Stress, and Perceived Medical Errors in Singapore Psychiatry Residents. Academic Psychiatry. 2021;45: 169–173. doi: 10.1007/s40596-020-01376-w 33409942

[6] Farhangi P, Khajehnasiri F. The Prevalence of Depression, Anxiety, and Stress Among Medical Residents: A Cross-Sectional Study in Iran. Acta Med Iran. 2020.

[7] Sadiq MS, Morshed NM, Rahman W, Chowdhury NF, Yasir Arafat SM, Mullick MSI. Depression, anxiety, stress among postgraduate medical residents: A Cross sectional Observation in Bangladesh. Iran J Psychiatry. 2019;141. doi: 10.18502/ijps.v14i3.1320 31598121 PMC6778600

[8] Dyrbye LN, West CP, Satele D, Boone S, Tan L, Sloan J, et al. Burnout among u.s. medical students, residents, and early career physicians relative to the general u.s. population. Academic Medicine. 2014;89. doi: 10.1097/ACM.0000000000000134 24448053

[9] Fahrenkopf AM, Sectish TC, Barger LK, Sharek PJ, Lewin D, Chiang VW, et al. Rates of medication errors among depressed and burnt out residents: Prospective cohort study. BMJ. 2008;336. doi: 10.1136/bmj.39469.763218.BE 18258931 PMC2258399

[10] Ishikawa M. Relationships between overwork, burnout and suicidal ideation among resident physicians in hospitals in Japan with medical residency programmes: a nationwide questionnaire-based survey. BMJ Open. 2022;12. doi: 10.1136/bmjopen-2021-056283 35273058 PMC8915267

[11] Nobleza D, Hagenbaugh J, Blue S, Skahan S, Diemer G. Resident Mental Health Care: a Timely and Necessary Resource. Academic Psychiatry. 2021;45. doi: 10.1007/s40596-021-01422-1 33660238

[12] Mata DA, Ramos MA, Bansal N, Khan R, Guille C, Di Angelantonio E, et al. Prevalence of Depression and Depressive Symptoms Among Resident Physicians A Systematic Review and Meta-analysis. JAMA. 2015;314: 2373. doi: 10.1001/jama.2015.15845 26647259 PMC4866499

[13] World Health Organization. Suicide worldwide in 2019: global health estimates. World Health Organization,Geneva. 2021.

[14] Zhang J, Liu X, Fang L. Combined effects of depression and anxiety on suicide: A case-control psychological autopsy study in rural China. Psychiatry Res. 2019;271. doi: 10.1016/j.psychres.2018.11.010 30529321 PMC6382523

[15] Reyes C, Santana V, Arocha G, Martínez N, Almonte K. Prevalence of depressive symptoms and suicide risk among medical residents. European Psychiatry. 2022;65: S552–S552. doi: 10.1192/J.EURPSY.2022.1412

[16] Elbatrawy AN, Mahmoud DA, Mohamed MY, Mohamed MM. Depression and suicidal ideations in relation to occupational stress in a sample of Egyptian medical residents. QJM: An International Journal of Medicine. 2020;113. doi: 10.1093/qjmed/hcaa054.020 35166153

[17] Dyrbye LN, Thomas MR, Massie FS, Power D V., Eacker A, Harper W, et al. Burnout and suicidal ideation among U.S. medical students. Ann Intern Med. 2008;149. doi: 10.7326/0003-4819-149-5-200809020-00008 18765703

[18] Laramée J, Kuhl D. Suicidal ideation among family practice residents at the University of British Columbia. Canadian Family Physician. 2019. 31604743 PMC6788667

[19] Yaghmour NA, Brigham TP, Richter T, Miller RS, Philibert I, Baldwin DC, et al. Causes of death of residents in ACGME-Accredited programs 2000 through 2014: Implications for the learning environment. Academic Medicine. 2017;92. doi: 10.1097/ACM.0000000000001736 28514230 PMC5483979

[20] Chahal S, Nadda A, Govil N, Gupta N, Nadda D, Goel K, et al. Suicide deaths among medical students, residents and physicians in India spanning a decade (2010–2019): An exploratory study using on line news portals and Google database. International Journal of Social Psychiatry. 2022;68. doi: 10.1177/00207640211011365 33904322

[21] Shoib S, Nagendrappa S, Grigo O, Rehman S, Ransing R. Factors associated with COVID-19 outbreak-related suicides in India. Asian Journal of Psychiatry. 2020. doi: 10.1016/j.ajp.2020.102223 32574941

[22] Neshan M, Malakouti SK, Kamalzadeh L, Makvand M, Campbell A, Ahangari G. Alterations in T-Cell Transcription Factors and Cytokine Gene Expression in Late-Onset Alzheimer’s Disease. Journal of Alzheimer’s Disease. 2022;85. doi: 10.3233/JAD-210480 34864659

[23] Rotenstein LS, Ramos MA, Torre M, Bradley Segal J, Peluso MJ, Guille C, et al. Prevalence of depression, depressive symptoms, and suicidal ideation among medical students a systematic review and meta-analysis. JAMA—Journal of the American Medical Association. 2016. doi: 10.1001/jama.2016.17324 27923088 PMC5613659

[24] Nazari S, Ghazvini H, Tamijani SMS, Rafaiee R. Increased Suicide of Iranian Resident Physicians in the COVID-19 Pandemic. Int J High Risk Behav Addict. 2022;11. doi: 10.5812/ijhrba.117651

[25] Ghaffari Elnaz. Suicide among Iranians doctors. International Journal of Science and Research Archive. 2023;9. doi: 10.30574/ijsra.2023.9.2.0607

[26] Saeed F, Shoib S, Esmaeeli ST. Physician suicide during the COVID-19 pandemic in Iran. Dusunen Adam—The Journal of Psychiatry and Neurological Sciences. 2021. doi: 10.14744/DAJPNS.2021.00151

[27] Arafat SMY, Ahmad A, Saeed A, Feizi O, Saeed F, Menon V, et al. Quality of Online Media Reporting of Suicidal Behavior in Iran during COVID-19 pandemic in Reference to the World Health Organization Guidelines. GLOBAL PSYCHIATRY ARCHIVES. 2022;5. doi: 10.52095/gpa.2022.4883.1047

[28] Ministry of Health and Medical Education. [cited 13 Jan 2024]. Available: https://irangov.ir/ministry-of-health-and-medical-education.

[29] Beck AT, Kovacs M, Weissman A. Assessment of suicidal intention: The Scale for Suicide Ideation. J Consult Clin Psychol. 1979;47. doi: 10.1037//0022-006x.47.2.343 469082

[30] Pinninti N, Steer RA, Rissmiller DJ, Nelson S, Beck AT. Use of the Beck Scale for Suicide Ideation with psychiatric inpatients diagnosed with schizophrenia, schizoaffective, or bipolar disorders. Behaviour Research and Therapy. 2002;40. doi: 10.1016/s0005-7967(02)00002-5 12296492

[31] Čebašek-Travnik Z. Review of the manual for the Beck scale for suicide ideation BSS. Psiholoska Obzorja. 2014.

[32] Baek IC, Jo S, Kim EJ, Lee GR, Lee DH, Jeon HJ. A Review of Suicide Risk Assessment Tools and Their Measured Psychometric Properties in Korea. Frontiers in Psychiatry. 2021. doi: 10.3389/fpsyt.2021.679779 34239463 PMC8258346

[33] Esfahani M, Hashemi Y, Alavi K. Psychometric assessment of beck scale for suicidal ideation (BSSI) in general population in Tehran. Med J Islam Repub Iran. 2015;29. 26793659 PMC4715388

[34] Lovibond SH, Lovibond PF. Manual for the Depression Anxiety Stress Scales. Psychology Foundation of Australia. 1995.

[35] Zanon C, Brenner RE, Baptista MN, Vogel DL, Rubin M, Al-Darmaki FR, et al. Examining the Dimensionality, Reliability, and Invariance of the Depression, Anxiety, and Stress Scale–21 (DASS-21) Across Eight Countries. Assessment. 2021;28. doi: 10.1177/1073191119887449 31916468

[36] Kakemam E, Navvabi E, Albelbeisi AH, Saeedikia F, Rouhi A, Majidi S. Psychometric properties of the Persian version of Depression Anxiety Stress Scale-21 Items (DASS-21) in a sample of health professionals: a cross-sectional study. BMC Health Serv Res. 2022;22. doi: 10.1186/s12913-022-07514-4 35078477 PMC8789546

[37] Malakouti SK, Nojomi M, Poshtmashadi M, Hakim Shooshtari M, Mansouri Moghadam F, Rahimi-Movaghar A, et al. Integrating a suicide prevention program into the primary health care network: A field trial study in Iran. Biomed Res Int. 2015;2015. doi: 10.1155/2015/193729 25648221 PMC4306260

[38] Hakim Shooshtari M, Malakouti SK, Panaghi L, Mohseni S, Mansouri N, Movaghar AR. Factors associated with suicidal attempts in Iran: A systematic review. Iranian Journal of Psychiatry and Behavioral Sciences. 2016. doi: 10.17795/ijpbs-948 27284284 PMC4898752

[39] Khosravi M, Kasaeiyan R. The relationship between neuroticism and suicidal thoughts among medical students: Moderating role of attachment styles. J Family Med Prim Care. 2020;9. doi: 10.4103/jfmpc.jfmpc_1200_19 32984107 PMC7491799

[40] Sadeghian MH, Etesam F, Nakhostin-Ansari A, Akbarpour S, Akhlaghi M. Association Between Hopelessness And Suicidal Ideation In Iranian Medical Students: A Cross-Sectional Study. Health Psychol Res. 2021;9. doi: 10.52965/001C.27579 35106399 PMC8801550

[41] Menon NK, Shanafelt TD, Sinsky CA, Linzer M, Carlasare L, Brady KJS, et al. Association of Physician Burnout with Suicidal Ideation and Medical Errors. JAMA Netw Open. 2020;3. doi: 10.1001/jamanetworkopen.2020.28780 33295977 PMC7726631

[42] Sousa GS de, Ramos BMD, Tonaco LAB, Reinaldo AMDS, Pereira MO, Botti NCL. Factors associated with suicide ideation of healthcare university students. Rev Bras Enferm. 2021;75Suppl. 3. doi: 10.1590/0034-7167-2020-0982 34852151

[43] Dabiran S, Nabaei B, Ghorbany M, Khajehnasiri F. Self-Reported Assessment of Health Status and Psychological Condition among Hospital Medical Residents in Iran. Health N Hav. 2015;07. doi: 10.4236/health.2015.77097

[44] Khorvash F, Askari G, Vesal S, Mehrbod N, Ghasemi H, Fatehizade M, et al. Investigating the anxiety level in iranian medical residents in 2010–2011. Int J Prev Med. 2013;4. 23776745 PMC3678239

[45] Zohn JH, Hovis S. The impact of the global COVID-19 pandemic on risk factors for suicide in healthcare workers: A narrative review. Journal of Clinical Nursing. 2023. doi: 10.1111/jocn.16651 36797214

[46] Iran Economic Monitor, Spring 2022. Economic Updates and Modeling. World Bank; 2022. doi: 10.1596/37792

[47] Masoomi M, Hosseinikolbadi S, Saeed F, Sharifi V, Jalali Nadoushan AH, Shoib S. Stigma as a barrier to suicide prevention efforts in Iran. Front Public Health. 2023;10. doi: 10.3389/fpubh.2022.1026451 36699938 PMC9868841

[48] Shoib S, Chandradasa M, Saeed F, Armiya’u AY, Roza TH, Ori D, et al. Suicide, Stigma and COVID-19: A Call for Action From Low and Middle Income Countries. Front Psychiatry. 2022;13. doi: 10.3389/fpsyt.2022.894524 35845441 PMC9283681

[49] Shooshtary MH, Malakouti SK, Bolhari J, Nojomi M, Poshtmashhadi M, Amin SA, et al. Community study of suicidal behaviors and risk factors among Iranian adults. Archives of Suicide Research. 2008;12. doi: 10.1080/13811110701857475 18340596

[50] Hassanian-Moghaddam H, Zamani N. Suicide in Iran: The facts and the figures from nationwide reports. Iran J Psychiatry. 2017;12. 28496505 PMC5425355

[51] Monzavi SM, Dadpour B, Shahraki K, Nemati M. Internal Medicine Residency Program in Iran: Exclusive Features and an International Comparison. Future of Medical Education Journal. 2017;7: 24–28. doi: 10.22038/fmej.2017.24129.1151

[52] Gharebaghi R, Heidary F, Pourezzat AA. Serial Deaths of Young Trainee Physicians in Iran During COVID-19 Pandemic; Messages to Policy Makers. Frontiers in Health Services. 2022;2. doi: 10.3389/frhs.2022.777065 36925768 PMC10012792

[53] Dutheil F, Aubert C, Pereira B, Dambrun M, Moustafa F, Mermillod M, et al. Suicide among physicians and health-care workers: A systematic review and meta-analysis. PLoS One. 2019;14. doi: 10.1371/journal.pone.0226361 31830138 PMC6907772

[54] Sen S, Kranzler HR, Krystal JH, Speller H, Chan G, Gelernter J, et al. A prospective cohort study investigating factors associated with depression during medical internship. Arch Gen Psychiatry. 2010;67. doi: 10.1001/archgenpsychiatry.2010.41 20368500 PMC4036806

[55] Kamalzadeh L, de Filippis R, El Hayek S, Heidari Mokarar M, Jatchavala C, Koh EBY, et al. Impact of stigma on the placement of mental health facilities: insights from early career psychiatrists worldwide. Front Psychiatry. 2023;14. Available: https://www.frontiersin.org/articles/10.3389/fpsyt.2023.1307277. doi: 10.3389/fpsyt.2023.1307277 38193134 PMC10773878

[56] Adiukwu F, Kamalzadeh L, Pinto da Costa M, Ransing R, de Filippis R, Pereira-Sanchez V, et al. The grief experience during the COVID-19 pandemic across different cultures. Annals of General Psychiatry. 2022. doi: 10.1186/s12991-022-00397-z 35701763 PMC9196145

[57] ML G, RN S, CA B. Depression and suicide among physician trainees: Recommendations for a national response. JAMA Psychiatry. 2015;72. doi: 10.1001/jamapsychiatry.2014.3050 25738529

[58] Frajerman A. Which interventions improve the well-being of medical students? A review of the literature. Encephale. 2020;46. doi: 10.1016/j.encep.2019.09.004 31767254

[59] Shalbafan M, Najarzadegan M, Soraya S, Rashedi V, Najafian R, Ahmadkhaniha H, et al. Validity and Reliability of the Persian Version of Internet Sex Screening Test in Iranian Medical Students. Sex Addict Compulsivity. 2019;26: 361–370. doi: 10.1080/10720162.2019.1645060

[60] Kamalzadeh L, Nayeri V, Soraya S, Shariat S V, Alavi K. Determining Test-Retest Reliability and Internal Consistency of the Persian Version of Personality Inventory For Diagnostic and Statistical Manual of Mental Disorders-5th Edition (PID-5) Among Medical Students and Patients With Psychiatric Disorders. Journal of Isfahan Medical School (IUMS). 2016;34: 901–907. Available: https://www.sid.ir/en/Journal/ViewPaper.aspx?ID=528108.

[61] Daré LO, Bruand PE, Gérard D, Marin B, Lameyre V, Boumédiène F, et al. Co-morbidities of mental disorders and chronic physical diseases in developing and emerging countries: A meta-analysis. BMC Public Health. 2019;19. doi: 10.1186/s12889-019-6623-6 30866883 PMC6417021

