# Supplementary material for: Factors associated with suicidal ideation among medical residents in Tehran during the COVID-19 pandemic: A multicentric cross-sectional survey
Source: PLoS One. 2024 Mar 15;19(3):e0300394. doi: 10.1371/journal.pone.0300394 (PMC10942077; doi:10.1371/journal.pone.0300394)
Supplement: S1 Checklist — (DOCX) [file pone.0300394.s001.docx]

STROBE Statement—checklist of items that should be included in reports of observational studies

|  | Item No. | Recommendation | Page  No. | Relevant text from manuscript |
| --- | --- | --- | --- | --- |
| **Title and abstract** | 1 | (*a*) Indicate the study’s design with a commonly used term in the title or the abstract | 1 | “Factors associated with suicidal ideation among medical residents in Iran during the COVID-19 pandemic: a multicentric cross-sectional survey” |
|  |  | (*b*) Provide in the abstract an informative and balanced summary of what was done and what was found | 3 | **Abstract**  Background: The mental well-being of medical residents is a critical concern, given the demanding and challenging nature of their training. This study aimed to investigate the factors associated with suicidal ideation among medical residents in Iran during the COVID-19 pandemic.  Methods: This study conducted a cross-sectional online survey among medical residents in various specialties in Tehran, Iran, amidst the COVID-19 pandemic. Suicidal ideation was assessed using the Beck Scale for Suicidal Ideation (BSSI), while depression, anxiety, and stress were measured using the DASS-21. It also collected demographic and clinical data from the participants. The data were analyzed using descriptive statistics, the Chi-square test, and multiple linear regression to examine the prevalence and determinants of suicidal ideation among medical residents.  Results: The study enrolled 353 medical residents and found that 34.3% of them had suicidal ideation, with 10.2% indicating a high risk. The study also found high levels of depression, anxiety, and stress among the participants. The variables that significantly predicted suicidal ideation were history of alcohol/substance use, family history of suicide attempts, personal history of suicide attempts, history of self-mutilation, number of shifts in a month, death of close persons because of COVID-19, income, and depression. Depression was the strongest predictor of suicidal ideation.  Conclusion: These findings underscore the urgent need for effective interventions and support systems to address the mental health needs of medical residents in Iran. The strategies should prioritize destigmatizing mental health, promoting access to mental health services, fostering a supportive training environment, and enhancing income opportunities. |
| Introduction | | | |  |
| Background/rationale | 2 | Explain the scientific background and rationale for the investigation being reported | 5-6 | **Introduction**  The mental well-being of medical residents is a critical concern due to the demanding and challenging nature of their training, which can have profound implications for patient care(1). Medical residency represents a pivotal phase in physicians' careers, characterized by long working hours, sleep deprivation, heightened stress levels, social deprivation, and exposure to emotionally taxing circumstances. These factors can cause considerable psychological distress and may increase the risk of developing psychiatric disorders(2).  Research conducted in low- and middle-income countries has shown a notable prevalence of depression and anxiety among residents in various specialized fields, ranging from 11% to 65%(3–7). While residents in these countries face additional challenges, such as limited healthcare resources, low income, and disparities in healthcare distribution(4), it is crucial to recognize that high rates of mental health problems in medical residents are not exclusive to developing countries. Studies conducted in high-income countries have also reported similarly elevated rates of depressive and anxiety disorders among medical residents, ranging from 7% to 43%(8–11). A comprehensive meta-analysis incorporating data from 54 studies worldwide revealed an overall prevalence of depressive symptoms of 28.8% among 17,560 resident physicians(12). These rates significantly exceed the lifetime prevalence of depression in the general adult population worldwide, which stands at 5%, as verified by the World Health Organization (WHO)(13).  Depression, particularly when comorbid with anxiety disorders, can lead to severe outcomes such as suicidal thoughts and behaviors(14). Several studies have documented the occurrence of suicidal ideation among medical residents, with prevalence rates ranging from 4 to 35 percent(15–18). According to the Accreditation Council for Graduate Medical Education (ACGME), suicide is the second leading cause of resident deaths in the USA, with the majority of suicides occurring during the first two years of training(19). Disturbingly, reports from India indicate 105 suicide-related deaths among residents between 2010 and 2019, with academic stress and harassment identified as significant risk factors(20,21). Various factors have been proposed to explain the high suicide rate among residents, including long and unpredictable work shifts, sleep deprivation, burnout and impaired cognitive performance, high-stress situations (e.g., life and death emergencies), and easy access to means of self-harm(15,17,22).  The predictors of suicidal thoughts and behaviors among medical residents may vary across different regions, cultures, and contexts. In recent years, Iran has witnessed alarming cases of suicide among medical residents, some occurring in succession within a short period, indicating a potential increase compared to previous decades. Several hypotheses have been suggested as potential causes, including heavy workloads, particularly during the COVID-19 pandemic, high patient care loads, financial pressures, the stigma of mental disorders, and inadequate support from authorities(23–25). However, a comprehensive assessment of the dimensions of this issue is yet to be conducted, especially since cases of resident suicide continue to be reported even after the subsiding of the COVID-19 pandemic. |
| Objectives | 3 | State specific objectives, including any prespecified hypotheses | 6 | Hence, this study aimed to shed light on factors associated with suicidal ideation among medical residents in Iran. Understanding the unique challenges medical residents face in Iran is crucial for developing effective interventions and support systems to address their mental health needs. |
| Methods | | | |  |
| Study design | 4 | Present key elements of study design early in the paper | 6-7 | **Study Design**  This cross-sectional survey aimed to explore the factors associated with suicidal ideation among medical residents from different specialties in Tehran, the capital of Iran, during the COVID-19 pandemic. The survey used a combination of standardized scales and demographic questions to measure the variables of interest. |
| Setting | 5 | Describe the setting, locations, and relevant dates, including periods of recruitment, exposure, follow-up, and data collection | 7 | **Setting**  The survey was conducted online using a Google form questionnaire distributed via email or WhatsApp groups to the residents of medical universities in Tehran. The data collection period was from 1 April to 10 March 2022, between the third and fourth waves of the COVID-19 pandemic in Iran. |
| Participants | 6 | (*a*) *Cohort study*—Give the eligibility criteria, and the sources and methods of selection of participants. Describe methods of follow-up  *Case-control study*—Give the eligibility criteria, and the sources and methods of case ascertainment and control selection. Give the rationale for the choice of cases and controls  *Cross-sectional study*—Give the eligibility criteria, and the sources and methods of selection of participants | 7 | **Participants**  The study included medical residents from various specialized fields across Tehran Medical Universities, namely Tehran University of Medical Sciences (TUMS), Shahid Beheshti University of Medical Sciences (SBUMS), Iran University of Medical Sciences (IUMS), and University of Social Welfare and Rehabilitation Sciences (USWR), representing different stages of residency training. Based on their field of practice, they were categorized into three groups: procedural specialties (including general surgery, neurosurgery, gynecology, emergency medicine, otolaryngology, urology, Orthopedics, and ophthalmology), clinical specialties (compromising internal medicine, psychiatry, neurology, pediatrics, radio-oncology, sports medicine, social medicine, and cardiology), and diagnostic specialties (encompassing radiology, pathology, and nuclear medicine). The exclusion criteria for this study comprised individuals who did not provide informed consent to participate in the research and those who were absent from the teaching hospital for three months preceding the study. The sample size was determined through statistical power analysis, considering a population percentage of 50%, a margin of error of 0.05, and a confidence level of 95%. The recommended sample size was 351 participants. The snowball method was employed for sampling. |
|  |  | (*b*) *Cohort study*—For matched studies, give matching criteria and number of exposed and unexposed  *Case-control study*—For matched studies, give matching criteria and the number of controls per case | N/A | N/A |
| Variables | 7 | Clearly define all outcomes, exposures, predictors, potential confounders, and effect modifiers. Give diagnostic criteria, if applicable | 8 | **Measures**  The online questionnaire comprised a total of 64 items encompassing various domains, including demographic data, specialty, year of residency, suicidal ideation, having children, living arrangements, accommodation, history of psychiatric disorders, alcohol/substance use, family history of suicide attempts, personal history of suicide attempts, history of self-mutilation, number of shifts in a month, COVID-19 work exposure, death of close persons because of COVID-19, use of mental health services during COVID-19 pandemic, income, and components of the Beck Scale for Suicidal Ideation (BSSI), as well as items from the Depression, Anxiety, and Stress Scale-21 (DASS-21). |
| Data sources/ measurement | 8* | For each variable of interest, give sources of data and details of methods of assessment (measurement). Describe comparability of assessment methods if there is more than one group |  | **Beck Scale for Suicidal Ideation (BSSI)**  The Beck Scale for Suicidal Ideation (BSSI) is a widely recognized and valuable tool for assessing suicidal ideation and behavior. It was developed in 1979 by Aaron T. Beck and consists of a 19-item self-report questionnaire that measures three dimensions of suicidal ideation: severity, frequency, and intent. Each item is rated on a scale of 0 to 2, indicating the absence, mild presence, or strong presence of specific suicidal thoughts. The total BSSI score ranges from 0 to 38(26).  The first five items of the BSSI can be used to identify individuals with suicidal thoughts. If patients score zero on the Active Suicidal Desire item (#4) or the Passive Suicidal Desire item (#5), indicating no active or passive intention to die, they skip the remaining 14 items. Otherwise, the remaining 14 items are rated. Patients with active or passive ideation about killing themselves are considered suicide ideators(27).  There is no universally defined cut-off score for BSSI to determine suicide risk levels; however, studies have proposed various criteria based on empirical evidence and clinical judgment. In this regard, scores of 0 on all 19 BSSI items indicate no risk of suicide. Scores ranging from 0 to 5 indicate low risk, while scores of 6 and above indicate high risk(28).  Extensive research has substantiated the robust psychometric properties of the BSSI, demonstrating high internal consistency (Cronbach's alpha coefficients between 0.89 and 0.96) and satisfactory test-retest reliability (correlation coefficients above 0.80)(29). The Persian version of the BSSI, used in the present study, has shown favorable reliability and validity in previous investigations(30).  **DASS-21**  The DASS-21 is a screening instrument designed to assess symptoms of depression, anxiety, and stress experienced in the week leading up to the evaluation. It consists of three subscales (depression, anxiety, and stress), each comprising seven items. The final score for each subscale is calculated by summing the scores of the corresponding items, which are rated on a scale of 0 (does not apply to me at all) to 3 (applies to me very much or most of the time)(31). The DASS-21 has demonstrated good internal consistency and reliability, as validated in various languages and populations(32). In the context of Iranian health workers, Kakemam et al. (2019) confirmed that the Persian version of the DASS-21 is a valid and reliable tool for assessing depression, anxiety, and stress(33). |
| Bias | 9 | Describe any efforts to address potential sources of bias | Efforts to address potential sources of bias are described throughout the “Methods” section on Pages 6-9 | Efforts were made to address potential sources of bias in this study. Firstly, the survey included medical residents from different specialties across multiple medical universities in Tehran, which helped in capturing a diverse range of perspectives and reducing selection bias. Additionally, the sample size was determined using statistical power analysis, ensuring adequate representation, and minimizing sampling bias. The snowball sampling method was employed, which enabled participants to refer their colleagues, further enhancing the diversity of the sample. Furthermore, standardized scales and demographic questions were used to measure the variables of interest, reducing measurement bias. To analyze the data, SPSS software was used, and appropriate statistical tests were applied to examine associations and identify predictors of suicidal ideation. The level of significance was set at 0.05 for all tests, maintaining rigor in the analysis. |
| Study size | 10 | Explain how the study size was arrived at | 7 | The sample size was determined through statistical power analysis, considering a population percentage of 50%, a margin of error of 0.05, and a confidence level of 95%. The recommended sample size was 351 participants. The snowball method was employed for sampling. |

Continued on next page

| Quantitative variables | 11 | Explain how quantitative variables were handled in the analyses. If applicable, describe which groupings were chosen and why | 9 | Descriptive statistics such as frequency and percentage were used to summarize the characteristics of the participants and their scores on the BSSI and the DASS-21. |
| --- | --- | --- | --- | --- |
| Statistical methods | 12 | (*a*) Describe all statistical methods, including those used to control for confounding | 9-10 | The data were analyzed using SPSS software version 25. Descriptive statistics such as frequency and percentage were used to summarize the characteristics of the participants and their scores on the BSSI and the DASS-21. A Chi-square test was used to examine the association between suicidal ideation and other clinical and demographic variables. A multiple linear regression analysis was used to identify the predictors of suicide ideation among the variables that were significantly associated with suicidal ideation in the Chi-square test. The level of significance was set at 0.05 for all tests. |
|  |  | (*b*) Describe any methods used to examine subgroups and interactions | N/A | N/A |
|  |  | (*c*) Explain how missing data were addressed | 10 | Cases with missing data were entirely excluded from the analysis. |
|  |  | (*d*) *Cohort study*—If applicable, explain how loss to follow-up was addressed  *Case-control study*—If applicable, explain how matching of cases and controls was addressed  *Cross-sectional study*—If applicable, describe analytical methods taking account of sampling strategy | N/A | N/A |
|  |  | (*e*) Describe any sensitivity analyses | N/A | N/A |
| Results | | | | |
| Participants | 13* | (a) Report numbers of individuals at each stage of study—eg numbers potentially eligible, examined for eligibility, confirmed eligible, included in the study, completing follow-up, and analysed | 10 | Out of the total of 359 residents who responded to the survey, six residents did not complete it. As a result, the final number of participants included in the study was 353. |
|  |  | (b) Give reasons for non-participation at each stage | 10 | Out of the total of 359 residents who responded to the survey, six residents did not complete it. As a result, the final number of participants included in the study was 353. |
|  |  | (c) Consider use of a flow diagram | A flow diagram was not utilized in the study. | A flow diagram was not utilized in the study. |
| Descriptive data | 14* | (a) Give characteristics of study participants (eg demographic, clinical, social) and information on exposures and potential confounders | 10-12 | The results show that most of the participants were female (75.9%), aged 30-34 years (50.1%), married (58.1%), clinical specialists (67.7%), in their first year of residency (31.4%), without children (84.4%), living with relatives (70%), renting or owning a home (91%), without a history of psychiatric disorders (63.5%), alcohol/substance use (77.3%), family history of suicide attempts (85.6%), personal history of suicide attempts (94.6%) or self-mutilation (94.9%). The average number of shifts in a month was 0-7 (49.6%), and the level of COVID-19 work exposure was moderate (43.1%). Most of the participants had a history of COVID-19 infection (74%) but were not admitted due to COVID-19 infection (95.2%) or the death of close persons because of COVID-19 (56.9%). About one-third of the participants used mental health services during the COVID-19 pandemic (33%) and had an income of less than 6 million Tomans (64%).  The BSSI scores indicated that 65.7% of the participants had no suicidal ideation, 24.1% had low risk suicidal thoughts, and 10.2% had high risk suicidal ideation. The overall prevalence of suicidal ideation was 34.3%. The levels of depression, anxiety, and stress were also high among the participants, with more than half reporting moderate to very severe depression (56.6%), anxiety (51.3%), and stress (45.3%).". Further demographic and clinical characteristics can be found in Table 1. |
|  |  | (b) Indicate number of participants with missing data for each variable of interest | N/A | N/A |
|  |  | (c) *Cohort study*—Summarise follow-up time (eg, average and total amount) | N/A | N/A |
| Outcome data | 15* | *Cohort study*—Report numbers of outcome events or summary measures over time | N/A | N/A |
|  |  | *Case-control study—*Report numbers in each exposure category, or summary measures of exposure | N/A | N/A |
|  |  | *Cross-sectional study—*Report numbers of outcome events or summary measures | 10-17 | Tables 1-3 |
| Main results | 16 | (*a*) Give unadjusted estimates and, if applicable, confounder-adjusted estimates and their precision (eg, 95% confidence interval). Make clear which confounders were adjusted for and why they were included | N/A | N/A |
|  |  | (*b*) Report category boundaries when continuous variables were categorized | N/A | N/A |
|  |  | (*c*) If relevant, consider translating estimates of relative risk into absolute risk for a meaningful time period | N/A | N/A |

Continued on next page

| Other analyses | 17 | Report other analyses done—eg analyses of subgroups and interactions, and sensitivity analyses | N/A | N/A |
| --- | --- | --- | --- | --- |
| Discussion | | | | |
| Key results | 18 | Summarise key results with reference to study objectives | 17-20 | **Discussion**  This study examined the factors associated with suicidal ideation among medical residents in Iran during the third and fourth waves of the COVID-19 pandemic. The findings revealed that a significant proportion of participants (34.3%) reported having suicidal ideas, with 10.2% indicating high-risk suicidal thoughts. These rates were higher than those reported in the general population of Iran (12.7-14%) and in medical students (7-26%) in previous studies(34–37). The prevalence of suicidal thoughts among medical residents in this study also surpassed the rates reported in recent studies conducted in other countries(10,38,39).  The study further explored the levels of depression, anxiety, and stress experienced by the participants. The findings revealed that more than 50% of the medical residents reported moderate to severe symptoms… |
| Limitations | 19 | Discuss limitations of the study, taking into account sources of potential bias or imprecision. Discuss both direction and magnitude of any potential bias | 20-21 | The study has several limitations that should be acknowledged. First, the study used a cross-sectional design, which limits the causal inference between the variables. Longitudinal studies are needed to examine the temporal relationship between suicidal thoughts and other factors among medical residents. Second, the study relied on self-report measures, which may be subject to recall and social desirability biases. Objective measures such as clinical interviews are needed to validate the self-report measures. Third, the study used a sample of medical residents from one city in Iran, which limits the generalizability of the findings to other populations or settings. Representative samples from different regions or cities are needed to examine the cross-cultural differences in suicidal thoughts among medical residents. The fourth limitation of our study is the omission of additional risk factors that could potentially influence the outcomes. For instance, we did not assess the impact of personality traits, marital discord, conflicts with family or colleagues, and the potential protective role of social support and religious beliefs. These factors have been identified in previous research as potential contributors to mental health outcomes(53,54) and could have provided a more comprehensive understanding of the associations observed in our study. Future studies should consider incorporating these factors to obtain a more nuanced perspective on the influences on mental health in this context. |
| Interpretation | 20 | Give a cautious overall interpretation of results considering objectives, limitations, multiplicity of analyses, results from similar studies, and other relevant evidence | 20-21 | The study's findings have significant implications for policy and practice. Firstly, they emphasize the urgent need to establish accessible, affordable, and high-quality mental health care and support for healthcare workers, particularly during critical situations like the COVID-19 pandemic. This is especially crucial for individuals with a history or current diagnosis of mental health problems or suicidal thoughts. Secondly, the findings highlight the importance of raising awareness and reducing mental health stigma among medical residents. Equipping them with coping skills and establishing peer support networks can help them manage the stress and challenges inherent in their profession. Additionally, there is a call for improving the working conditions and environment of medical residents, including workload reduction, ensuring their safety, and enhancing supervision and feedback mechanisms. Lastly, the study suggests that mental health education and promotion should be integrated into training programs, with ongoing monitoring and intervention for students and residents at risk of suicide.  Conclusion  The elevated occurrence of suicide among medical residents in Iran can be attributed to various factors, with depression being the most prominent predictor. These results underscore the pressing need for immediate intervention to tackle the mental health crisis. It is crucial to enhance support systems, offer comprehensive education and training, reduce work hours, and combat the stigma surrounding mental health problems. |
| Generalisability | 21 | Discuss the generalisability (external validity) of the study results | 20-21 | The study's findings have significant implications for policy and practice. Firstly, they emphasize the urgent need to establish accessible, affordable, and high-quality mental health care and support for healthcare workers, particularly during critical situations like the COVID-19 pandemic. This is especially crucial for individuals with a history or current diagnosis of mental health problems or suicidal thoughts. Secondly, the findings highlight the importance of raising awareness and reducing mental health stigma among medical residents. Equipping them with coping skills and establishing peer support networks can help them manage the stress and challenges inherent in their profession. Additionally, there is a call for improving the working conditions and environment of medical residents, including workload reduction, ensuring their safety, and enhancing supervision and feedback mechanisms. Lastly, the study suggests that mental health education and promotion should be integrated into training programs, with ongoing monitoring and intervention for students and residents at risk of suicide. |
| Other information | |  | | |
| Funding | 22 | Give the source of funding and the role of the funders for the present study and, if applicable, for the original study on which the present article is based | According to the PLOS ONE submission rules, funding information is not included in the manuscript. | - |

*Give information separately for cases and controls in case-control studies and, if applicable, for exposed and unexposed groups in cohort and cross-sectional studies.

**Note:** An Explanation and Elaboration article discusses each checklist item and gives methodological background and published examples of transparent reporting. The STROBE checklist is best used in conjunction with this article (freely available on the Web sites of PLoS Medicine at http://www.plosmedicine.org/, Annals of Internal Medicine at http://www.annals.org/, and Epidemiology at http://www.epidem.com/). Information on the STROBE Initiative is available at www.strobe-statement.org.
