# Supplementary material for: Factors associated with suicidal ideation among medical residents in Tehran during the COVID-19 pandemic: A multicentric cross-sectional survey
Source: PLoS One. 2024 Mar 15;19(3):e0300394. doi: 10.1371/journal.pone.0300394 (PMC10942077; doi:10.1371/journal.pone.0300394)
Supplement: Attachment — Submitted filename: comments to the authors.docx [file pone.0300394.s003.docx]

The authors have adequately addressed most of the previous review comments. It would be nice to address the following remaining concerns.

1. The authors did not confirm the authenticity of the respondents. This should be acknowledged as a weakness to this study in the limitations section.
2. In the calculation of the sample size, it was not clear if the assumed 50% was referring to suicidal ideation or attempt or both ideation and attempt. It was also not clear if the 50% referred to low or high risk of suicide. This needs to be clarified.
3. When addressing the comment on confounding factors, the authors stated that they omitted additional factors. These need to be indicated in the revised manuscript.
