# Supplementary material for: Factors associated with suicidal ideation among medical residents in Tehran during the COVID-19 pandemic: A multicentric cross-sectional survey
Source: PLoS One. 2024 Mar 15;19(3):e0300394. doi: 10.1371/journal.pone.0300394 (PMC10942077; doi:10.1371/journal.pone.0300394)
Supplement: Attachment — Submitted filename: Response to reviewers comments2.docx [file pone.0300394.s004.docx]

**Response to Reviewers Comments**

**PONE-D-23-19377R1**

**PLOS ONE**

**February 25, 2024**

Dear Editor,

We are writing to submit the revised version of our manuscript titled " Factors associated with suicidal ideation among medical residents in Tehran during the COVID-19 pandemic: a multicentric cross-sectional survey," with the submission ID PONE-D-23-19377R1, for consideration in PLOS ONE. We appreciate the insightful comments from Reviewer #2 and the opportunity to improve our manuscript accordingly.

In response to the comments received, we have made the following key revisions:

- Acknowledged the limitation regarding the non-verification of respondent authenticity in the limitations section (Page 22, Lines 354-356).
- Clarified the assumption of the 50% prevalence rate specifically referring to suicidal ideation, addressing both low and high risk of suicide, for our sample size calculation (Page 8, Lines 130-132).
- Detailed the omitted confounding factors that were not previously addressed in the manuscript (Page 22, Lines 348-353).

Furthermore, due to the modifications suggested by the reviewer, we have added a new reference, now listed as reference number 61, to support our revised discussions.

Please find attached a detailed point-by-point response to all comments (reviewers’ comments in black, our replies in blue). The changes are highlighted in yellow in the revised manuscript.

We believe these revisions have significantly strengthened our manuscript by clarifying critical aspects of our study design and findings. We are grateful for the reviewers’ constructive feedback, which has been instrumental in enhancing the quality and clarity of our work.

We thank you for considering our revised manuscript and look forward to the possibility of our work being published in PLOS ONE.

Sincerely,

Leila Kamalzadeh, M.D., Assistant Professor of Psychiatry.

Geriatric mental health research center, Department of Psychiatry, School of Medicine, Iran University of Medical Sciences (IUMS), Tehran, Iran, Email: Kamalzadeh.l@iums.ac.ir, lkamalzadeh@gmail.com, ORCID ID: https://orcid.org/0000-0002-2444-7195

**Reviewer #2**

The authors have adequately addressed most of the previous review comments. It would be nice to address the following remaining concerns.

| 1. The authors did not confirm the authenticity of the respondents. This should be acknowledged as a weakness to this study in the limitations section. | We have now addressed the concern regarding respondent authenticity in the limitations section, lines 354-356 on page 22. |
| --- | --- |
| 2. In the calculation of the sample size, it was not clear if the assumed 50% was referring to suicidal ideation or attempt or both ideation and attempt. It was also not clear if the 50% referred to low or high risk of suicide. This needs to be clarified. | We have clarified the assumption of the 50% prevalence rate as referring to suicidal ideation, not attempts, covering both low and high risk of suicide, on page 8, lines 130-132. |
| 3. When addressing the comment on confounding factors, the authors stated that they omitted additional factors. These need to be indicated in the revised manuscript. | We have included the omitted confounding factors in the revised manuscript, specifically addressed on page 22, lines 348-353. |
